# Merging of the Case 2 Regional Coast Colour and Maximum-Peak Height chlorophyll-a algorithms: validation and demonstration of satellite-derived retrievals across US lakes

**DOI:** 10.1007/s10661-021-09684-w

**Published:** 2022-02-14

**Authors:** Blake Schaeffer, Wilson Salls, Megan Coffer, Carole Lebreton, Mortimer Werther, Kerstin Stelzer, Erin Urquhart, Daniela Gurlin

**Affiliations:** 1 Office of Research and Development, US EPA, Durham, NC 27709 USA; 2 Oak Ridge Institute for Science and Education, US EPA, Durham, NC 27709 USA; 3 Brockmann Consult, Hamburg, Germany; 4 Earth and Planetary Observation Sciences, Biological and Environmental Sciences, Faculty of Natural Sciences, University of Stirling, Stirling, UK; 5 Science Systems and Applications, Inc, NASA Goddard Space Flight Center, Greenbelt, MD 20771 USA; 6 Wisconsin Department of Natural Resources, Madison, WI 53707 USA

**Keywords:** Satellite, Water quality, Chlorophyll, Lakes, Reservoirs, Trophic state

## Abstract

Water quality monitoring is relevant for protecting the designated, or beneficial uses, of water such as drinking, aquatic life, recreation, irrigation, and food supply that support the economy, human well-being, and aquatic ecosystem health. Managing finite water resources to support these designated uses requires information on water quality so that managers can make sustainable decisions. Chlorophyll-*a* (chl-*a*, µg L^−1^) concentration can serve as a proxy for phytoplankton biomass and may be used as an indicator of increased anthropogenic nutrient stress. Satellite remote sensing may present a complement to in situ measures for assessments of water quality through the retrieval of chl-*a* with in-water algorithms. Validation of chl-*a* algorithms across US lakes improves algorithm maturity relevant for monitoring applications. This study compares performance of the Case 2 Regional Coast Colour (C2RCC) chl-*a* retrieval algorithm, a revised version of the Maximum-Peak Height (MPH_(P)_) algorithm, and three scenarios merging these two approaches. Satellite data were retrieved from the MEdium Resolution Imaging Spectrometer (MERIS) and the Ocean and Land Colour Instrument (OLCI), while field observations were obtained from 181 lakes matched with U.S. Water Quality Portal chl-*a* data. The best performance based on mean absolute multiplicative error (MAE_mult_) was demonstrated by the merged algorithm referred to as C_15_−M_10_ (MAE_mult_ = 1.8, bias_mult_ = 0.97, *n* = 836). In the C_15_−M_10_ algorithm, the MPH_(P)_ chl-*a* value was retained if it was > 10 µg L^−1^; if the MPH_(P)_ value was ≤ 10 µg L^−1^, the C2RCC value was selected, as long as that value was < 15 µg L^−1^. Time-series and lake-wide gradients compared against independent assessments from Lake Champlain and long-term ecological research stations in Wisconsin were used as complementary examples supporting water quality reporting requirements. Trophic state assessments for Wisconsin lakes provided examples in support of inland water quality monitoring applications. This study presents and assesses merged adaptations of chl-*a* algorithms previously reported independently. Additionally, it contributes to the transition of chl-*a* algorithm maturity by quantifying error statistics for a number of locations and times.

**Supplementary information:**

The online version contains supplementary material available at 10.1007/s10661-021-09684-w.

## Introduction

Eutrophication threatens the sustainability of lake ecosystems, well-being (Cox et al., 2006; Wheeler et al., 2012), and economies (Dodds et al., 2009) of communities around the world (UNEP, 2007; Wilson & Fischetti, 2010). Whether naturally induced or driven by human activities, high nutrient levels pose risks to both the environment and human health (Peierls et al., 1991). Under the right conditions, algae and cyanobacteria can proliferate, outcompeting native aquatic flora and fauna and threatening aquatic ecosystems. Additionally, some cyanobacteria create compounds toxic to humans and livestock, leading to public health and socio-economic risks (Stroming et al., 2020). For these reasons, understanding eutrophication is crucial. Chlorophyll-*a* (chl-*a*) concentration is often the targeted water quality indicator for nutrient eutrophication (Schaeffer et al., 2012), as its presence in water tends to originate from algae and cyanobacteria growth responses to nutrient availability.

There are limitations with in situ measures of chl-*a*, including variable accuracy where error can be as high as 30–60% for fluorescence methods (Trees et al., 1985; Bianchi et al., 1995) and spatial–temporal representation from discrete samples does not reflect the larger system. However, in situ measures can characterize the vertical distribution of chl-*a* throughout the water column. Significant differences exist among the methods to analyze chl-*a* samples in the laboratory. Most frequently, fluorescence methods are used to analyze chl-*a*. Less frequently, chl-*a* samples are analyzed with high performance liquid chromatography (HPLC). HPLC analysis involves greater material costs, sample run-times, and technological training requirements. The error associated with HPLC measurements is lower than that associated with fluorescence methods since pigment compounds are physically separated and individually quantified (Trees et al., 1985). In addition to traditional in situ sampling of chl-*a*, satellite remote sensing can provide measures of optically related water quality characteristics, including derived measures of chl-*a*, in lakes, and reservoirs (Gitelson, 1992; IOCCG, 2018). The spatial and temporal resolution of satellite remote sensing can help reduce costs associated with traveling to sites, laboratory analysis, and staffing to support these activities. Papenfus et al. (2020) reported mean cloud free temporal resolution of United States (US) lakes was 184 days per year with one Sentinel-3 satellite. Satellite remote sensing may also be a cost-effective option for state, regional, or national assessments of lake water quality (Papenfus et al., 2020). However, there are several inherent challenges with using satellite remote sensing for inland water quality monitoring. First, the spatial resolution of satellite sensors is generally too coarse to resolve small water bodies and nearshore environments (Clark et al., 2017). Second, satellite measurements are retrieved primarily from the upper part of the water column and therefore do not represent dynamics below the surface. Third, not all necessary bio-geochemical measures can be derived from satellites. Finally, the temporal resolution can be impacted by cloud cover, which often limits the number of viable satellite images per year (Mercury et al., 2012). Given these constraints, satellite remote sensing and in situ measures offer complementary approaches to chl-*a* monitoring of inland water bodies.

Though substantial effort has been put forth to validate a variety of chl-*a* algorithms over the past several decades (Matthews, 2011; Neil et al., 2019; Pahlevan et al., 2020), there are relatively few studies that do so, both at a fine spatial resolution and across a broad spatial scale relevant for water management applications and decision-making efforts for the inland waters of the USA. Until such broad validations are performed, reliable satellite-derived chl-*a* remains restricted to individual water bodies with existing in situ data or to water bodies with specifically tuned chl-*a* algorithms. Broader validations of chl-*a* algorithms across US inland waters may improve understanding of satellite-derived measures for management applications (Schaeffer et al., 2013b). Several studies have validated chl-*a* algorithms; however, most of them investigate either a single waterbody or a small collection of water bodies.

Large-scale assessment of chl-*a* algorithm performance across water bodies is challenging due to the optical complexity of inland waters. The presence of optically significant constituents such as colored dissolved organic matter, algae, and sediments confounds the satellite signal making differentiation of chl-*a* difficult (Gitelson et al., 2008). Odermatt et al. (2012) present one of the most comprehensive reviews of chl-*a* algorithm approaches for optically complex waters. Topp et al. (2020) found that most studies focused on developing algorithms and validation with only recently improved data availability enabling operational remote sensing algorithms to improve the quantification of inland water quality. Filazzola et al. (2020) synthesized a database of in situ chl-*a* for > 10,000 freshwater lakes across 72 countries for potential satellite validation. Other databases are becoming readily available that may aid in satellite algorithm validation, such as the Water Quality Portal (WQP, Read et al., 2017) in the USA and the Lake Bio-optical Measurements and Matchup Data for Remote Sensing (LIMNADES) worldwide (Spyrakos et al., 2018). This study found as of August 2019 a *Web of Science* search using keywords “lake, satellite, algorithm, chlorophyll” returned 273 journal articles with 23% focused on the Great Lakes along the border of the USA and Canada and Lake Taihu, China. The limited larger studies across multiple lakes included a validation of 185 lakes across the globe and > 100 sites within the USA (Neil et al., 2019; Spyrakos et al., 2018). Sayers et al. (2015) derived chl-*a* for 80,012 lakes across the globe using data from 37 lakes as validation, with 20 in the USA. Odermatt et al. (2018) derived measures for 340 lakes with 24 lakes for validation. Wang et al. (2018) assessed global trophic status in over 2,000 large inland water bodies. Huovinen et al. (2014) included 50 lakes in South Africa, and Lesht et al. (2014) used 23 water reservoirs in Spain. Even these larger studies have limited validation across lake systems, especially in the USA.

The MEdium Resolution Imaging Spectrometer (MERIS) onboard the Envisat satellite and the Ocean and Land Colour Instrument (OLCI) onboard the Sentinel-3A and Sentinel-3B satellites have a spatial resolution of 300 m and provide the potential to resolve >2,000 of the largest lakes and reservoirs in the contiguous USA (CONUS) (Schaeffer et al., 2018a; Urquhart & Schaeffer, 2020); however, validation efforts of satellite-derived chl-*a* across US lakes are still fairly limited in these systems. Validation efforts are necessary to quantify the algorithm maturity in order to advance application readiness levels (ARLs) for stakeholders. Algorithm maturity (NASA, 2020) can be defined into three general levels: beta, provisional, and validated. The validated level includes four stages of maturity: (1) algorithm error statistics are estimated from a small number of measurements from select locations and times; (2) algorithm error statistics are estimated from a significant number of locations and times, with consistency compared against similar efforts representing a comprehensive representation of locations and times; (3) algorithm error is assessed with uncertainties well quantified and robust compared to reference data; and (4) validation results are systematically updated with new algorithm updates and as time expands. Most algorithm evaluations involving a single waterbody or small collection of water bodies fall into validation stage 1 maturity.

This study compares two different chl-*a* retrieval algorithms and three scenarios merging these two algorithms using satellite data from both Envisat MERIS and Sentinel-3A OLCI with field observations from 181 water bodies across CONUS matched from the US WQP. The objective of this study is to assess the performance of each algorithm across water bodies with a range of environmental and optical conditions to initiate the transition from algorithm validation stage 1 to stage 2. Results from this study can help determine the usability of each chl-*a* retrieval algorithm and will also allow for evaluation of water quality metrics at both a fine and a broad spatial scale. Satellite-derived chl-*a* can complement in situ water quality metrics that are reported in large-scale monitoring programs, such as the U.S. Environmental Protection Agency (EPA) National Lakes Assessment (NLA, U.S. EPA, 2011) and the U.S. EPA National Coastal Condition Assessment (NCCA, U.S. EPA, 2012), to allow for more frequent reporting than otherwise possible with field sampling alone. Further, the value of satellite-derived chl-*a* can be demonstrated through various ecological applications, such as classification of lake trophic state, to aid in general condition assessments, identify trends in water quality, and track the successes of restoration actions.

## Methods and data

### In situ validation data

In the USA, in situ discrete water samples are collected by several monitoring and research organizations, many of whom do not follow the same practices, formats, and description approaches. To address these inconsistencies, the U.S. Geological Survey (USGS), the U.S. EPA, and the National Water Quality Monitoring Council (NWQMC) developed the WQP (www.waterqualitydata.us, Read et al., 2017). The WQP was developed as a publicly accessible database to simplify dissemination of water quality data in the USA, with > 290 million total records and > 3 million records on ground, inland, and coastal waters. Monitoring is performed through samples taken by state, federal, or tribal projects. We compiled a validation dataset from the WQP including chl-*a* measurements for inland water bodies resolvable by MERIS (2002 through 2012) and OLCI (2016 through 2019). Resolvable lakes were defined as lakes with at least three water pixels remaining in the National Hydrography Dataset (NHD) Plus version 2.0 (McKay et al., 2012) polygon, after excluding pixels adjacent to the shoreline. Radiometric information such as remote sensing reflectance or inherent optical properties, typically used to constrain the use of in situ observations, are not part of the WQP. The availability of in situ measures was dependent on the organizations voluntarily uploading data to the WQP; thus, observation data may be delayed anywhere from months to years (Papenfus et al., 2020). This delay caused the number of available in situ match-ups with OLCI to be considerably lower than for MERIS.

Measurements of chl-*a* at Lake Champlain were obtained from the Lake Champlain Long-Term Water Quality and Biological Monitoring Project at https://dec.vermont.gov/watershed/lakes-ponds/monitor/lake-champlain using EPA method 445.0 from the Vermont Department of Environmental Conservation (Arar & Collins, 1997). Measurements from 2018 taken at 1-m depth were retained for analysis. Those outside the detection limit of the instrumentation were removed. While monitoring is conducted at 15 points throughout the lake, only measurements at most central locations in the lake were considered to avoid errors caused by adjacency effects in the satellite measurements.

In situ measures of chl-*a* at Lake Mendota, Lake Monona (Magnuson et al., 2020), and Trout Lake in Wisconsin were obtained from the North Temperate Lakes US Long-Term Ecological Research (LTER) Network (https://lter.limnology.wisc.edu; Magnuson et al., 2019, 2020). Measurements from 2018 taken at a 0–2-m depth range were retained for analysis. LTER measurements containing a quality flag were discarded.

### Validation quality assurance

The measurements provided by the WQP were not intended for satellite algorithm validation. Therefore, WQP data were filtered based on quality assurance criteria, detailed here, to ensure appropriate fidelity prior to use for validation with the satellite algorithms. For example, the WQP data contained several chlorophyll pigment types. Many phytoplankton pigments (e.g., chlorophyll-*b*, -*c*) are not distinguishable with broad band multi-spectral satellite algorithms in optically complex inland waters (Chase et al., 2017; Muller-Karger et al., 2018), and only in situ chl-*a* measurements were retained in the validation dataset. Different laboratory analytical identifiers provided information about the respective extraction and analysis methods to measure in situ chl-*a* concentrations. There were various analytical methods used, and no single document exists listing all methods. A majority of the in situ chl-*a* measurements were from standard fluorometric methods such as EPA method 445.0 (Arar & Collins, 1997).

Bailey and Werdell (2006) recommend using validation data within ± 3 h of the satellite overpass for ocean waters, whereas in lakes, Rusak et al. (2018) reported hourly to daily phytoplankton biomass variations influenced by wind speed and storm events. Therefore, a temporal restriction of ± 6 h was used between in situ data collection and satellite overpass to maximize the number of potential in situ measures matched with satellite observations while minimizing the complexities of bio-physical changes such as vertical and horizontal movement of phytoplankton within the water column. In situ samples without a time stamp were assigned a time of 12:00 p.m. local time to retrieve the satellite overpass of the same day.

In situ samples were filtered to those with depth measures of ≤ 2 m or labeled as “surface” in resolvable lakes to avoid influences of the bottom albedo on the retrieved reflectances (Albert & Mobley, 2003) and to represent the top of the water column. Most of the light detected by a typical satellite sensor originates near the water’s surface, down to a depth of about 2 m in clear water (Mishra et al., 2005) and < 2 m in more turbid waters (Wynne et al., 2010). Additionally, nearby land areas influence the optical signals retrieved, where top-of-atmosphere radiance contamination from neighboring land surfaces with brighter reflectances causes adjacency effects (Bulgarelli & Zibordi, 2018). Therefore, an in-lake spatial filter was applied to all in situ locations to reduce adjacency effects from surrounding land environments. Spatial filtering to resolvable lakes using the NHD limited in situ data to lakes with at least three water pixels remaining in the NHD lake polygons, after quality control flagging pixels adjacent to the shoreline (Urquhart & Schaeffer, 2020). MERIS and OLCI 300-m at-nadir pixel size limits resolvable lakes in the USA to 0.7% of total lakes as defined by the NHD (Clark et al., 2017). A land-waterbody mask was generated using the NHD (McKay et al., 2012). This land–water mask functioned as the base layer; two water pixels adjacent to land were flagged as mixed land–water pixels potentially experiencing adjacency effects, providing a 600-m in-lake buffer. The dataset obtained from the WQP was then spatially clipped by this buffer to discard every in situ location not surrounded by at least 8 complete neighboring pure water pixels. This quality filter step removed any mixed land–water shoreline pixels in a MERIS or OLCI scene. It also guaranteed that a considered in situ location was at least 600 m from shore. In situ measures were matched only with the single pixel (1 × 1 pixel array) where the discrete sample position was located.

### MERIS and OLCI satellite data

Satellite observations were obtained from MERIS from 2002 through 2012, as the MERIS mission formally ended in April 2012 due to instrument failure. The Copernicus program’s new series of Sentinel-3 OLCIs (Berger et al., 2012; Donlon et al., 2012) replaced the previous MERIS sensor. The Sentinel-3A OLCI launched in February 2016, and a single mission offers a revisit frequency of approximately 2–3 days with 300-m spatial resolution at nadir. Data is collected in 21 spectral bands with center wavelengths ranging from 400 to 1020 nm. While Sentinel-3B launched in April 2018, data was not publicly available until late 2019 and there were limited in situ data available for match-up from the WQP; therefore, only Sentinel-3A data are utilized in this study.

**Fig. 1:**
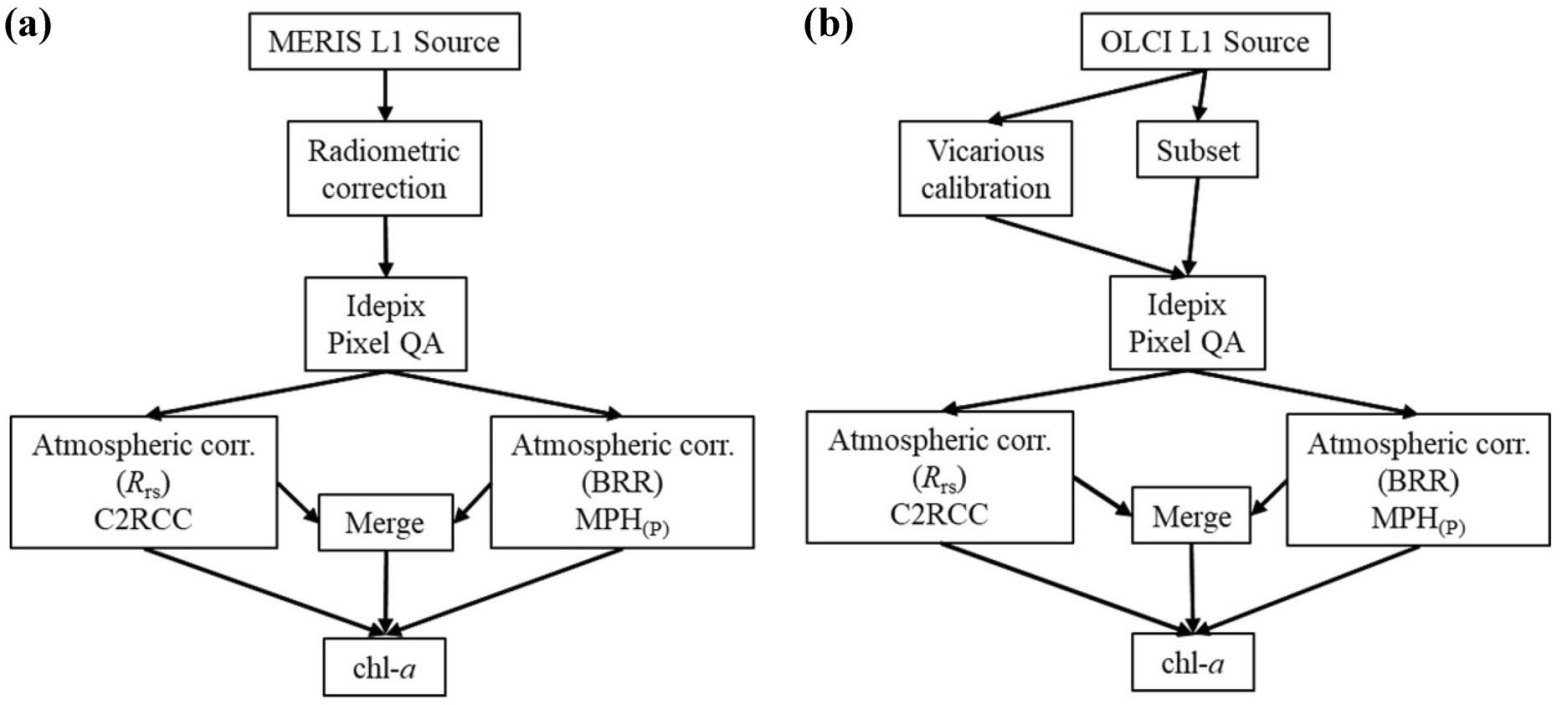
Processing workflow on the Calvalus Earth Observation processing cluster, publicly available in the Sentinel-3 Toolbox of ESA’s Sentinel Application Platform for **a** MERIS and **b** OLCI, where the algorithm merge workflow is detailed in Fig. 2

Producing temporally aggregated water quality parameters for a 12-year timeframe from Level-1 products with instrument and radiometric calibrations applied requires several methods in a processing chain (Fig. 1). This processing chain has been deployed on the Calvalus Earth Observation processing cluster of Brockmann Consult. Calvalus is a parallel processing system allowing for fast and iterative processing of satellite products (Fomferra et al., 2012). All processors used in this study are publicly available in the Sentinel-3 Toolbox of the European Space Agency’s (ESA) Sentinel Application Platform (SNAP; https://step.esa.int/main/toolboxes/snap/) and can be combined in bulk processing schemes.

It is critical to identify high-quality water pixels prior to implementing algorithms. Cloud or cloud shadow influenced pixels may negatively impact algorithm operations. Pure water pixels were retained using the Identification of pixel properties algorithm (IdePix), an open-source SNAP processor. IdePix performs the identification of clouds, cloud shadows, snow, ice, sun glint, and ambiguous mixed pixels. The processor consists of several linked algorithms: arithmetic expressions, spectral unmixing for pixel identification, and two back-propagation neural networks for Level-1B (calibrated, ortho-geolocated, and spatially re-gridded radiances) cloud identification (ESA, 2013). The satellite algorithms used in this study assume pure water reflection.

### Satellite algorithms for chl-*a*

In this study, we applied two distinct chl-*a* inversion algorithms, the Case 2 Regional Coast Colour (C2RCC) (Doerffer & Schiller, 2007) and Maximum-Peak Height (MPH) algorithms (Matthews & Odermatt, 2015; Matthews et al., 2012). C2RCC is based on independent neural networks trained with atmospheric and water-atmosphere radiative transfer simulation look-up tables. The first neural network corrected for atmospheric influences on top of atmosphere reflectance and calculated remote sensing reflectance. Remote sensing reflectance was then inverted by a subsequent neural network to derive chl-*a* concentration (Brockmann et al., 2016). C2RCC was validated in coastal marine waters using the Coast Colour Round Robin dataset (Nechad et al., 2015) with some lake in situ measurements and is available in ESA’s SNAP software (Brockmann et al., 2016).

The MPH algorithm uses bottom-of-Rayleigh reflectance (BRR) to derive chl-*a* in high biomass waters (Matthews & Odermatt, 2015; Matthews et al., 2012). The algorithm was designed with a peak position selector to search for the maximum radiance emitted. In the MPH procedure, a baseline was calculated over a large red to near infrared (NIR) range between the bands centered at 664 and 885 nm to determine the maximum peak intensity and position from the maximum radiance measured over 681, 709, or 753 nm. BRR was calculated by the Rayleigh processor incorporated in the SNAP MPH processor bundle. Based on computed BRR, the MPH was then calculated as follows:

$$\mathrm{MPH}={\mathrm{BRR}}_{\mathrm{max}}-{\mathrm{BRR}}_{664}-[\left({\mathrm{BRR}}_{885}-{\mathrm{BRR}}_{664}\right)\times \left(\frac{{\lambda }_{\mathrm{max}}-{\lambda }_{664}}{{\lambda }_{885}- {\lambda }_{664}}\right)]$$

where BRR_max_ and *λ*_max_ are the magnitude and position of the largest magnitude BRR from spectral bands centered at 681, 709, or 753 nm. Pitarch et al. (2017) updated the MPH algorithm (MPH_(P)_) to include new in situ calibration data for deriving chl-*a* from the MPH index values. The new regression for chl-*a* allowed for a transition between eukaryotes and cyanobacteria-dominant waters by combining both datasets, avoiding calibration with specific chl-*a* regressions for either eukaryote or cyanobacteria dominant waters. MPH_(P)_ chl-*a* was computed from MPH as follows:

$$\mathrm{Chla}\left[{\mathrm{MPH}}_{\mathrm{P}}\right]=848468 \times {\mathrm{MPH}}^{3}-72058\times {\mathrm{MPH}}^{2}+5515.7\times \mathrm{MPH}$$

Derived C2RCC and MPH_(P)_ chl-*a* were merged to achieve optimal measures across various inland water types. A recent study showed that C2RCC retrieved chl-*a* accurately in eukaryote dominant waters, turbid waters, and with chl-*a* concentrations typically < 10 µg L^−1^ (Kratzer & Plowey, 2021). Kravitz et al. (2020) reported the MPH minimum detection limit was potentially 1–5 µg L^−1^ with accuracy improving > 20 µg L^−1^.

**Fig. 2:**
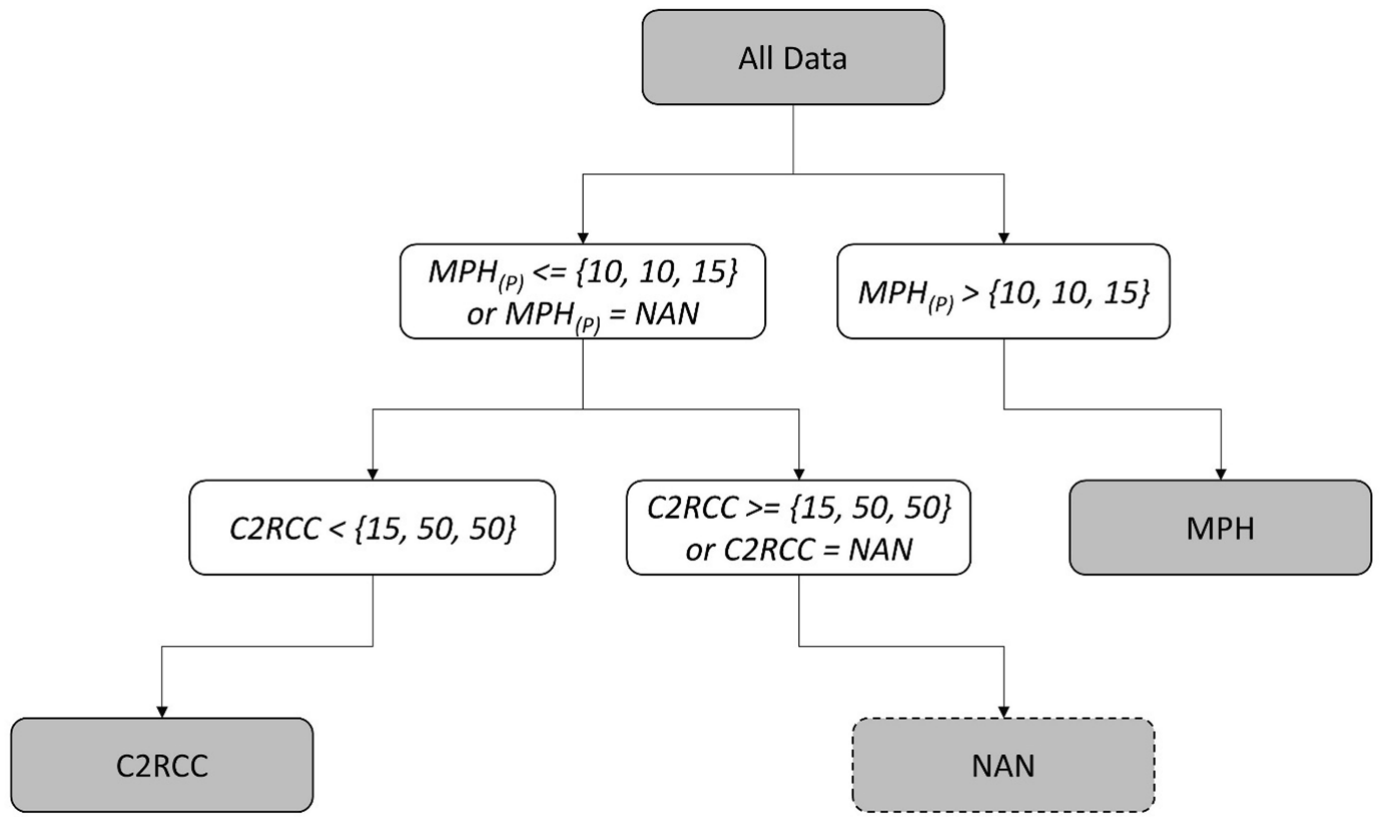
Workflow showing the logic applied to determine output values for the merged algorithms C_15_-M_10_, C_50_-M_10_, and C_50_-M_15_ based on the C2RCC and the MPH_(P)_ algorithms. NAN (not a number) indicates invalidity

In order to leverage each algorithm to perform optimally, merged algorithms were developed in which the MPH_(P)_ algorithm value was utilized in cases of relatively high chl-*a* values and the C2RCC algorithm for low chl-*a* values. The algorithms were combined such that the chl-*a* concentration resulting from MPH_(P)_ was retained if MPH_(P)_ chl-*a* was above a certain MPH_(P)_ minimum threshold value. If the chl-*a* concentration was below this value, the C2RCC result was selected if the pixel was valid and below a C2RCC maximum threshold value. If the pixel value was below MPH_(P)_ minimum threshold value and above the C2RCC maximum threshold, it was discarded. A schematic diagram showing the logical selection process of the merged algorithm values is shown in Fig. 2. To select optimal threshold values, all combinations of MPH_(P)_ and C2RCC threshold values were assessed to identify the one yielding the lowest error. Error was assessed through calculation of the mean absolute multiplicative error (MAE_mult_), a metric explained in the subsequent section. In addition to the merged algorithm using previously published optimal thresholds, two other merged algorithms were considered based on natural breaks that occurred in the in situ data set.

### Algorithm assessment

To assess the performance of each algorithm, single-pixel chl*-a* values were compared against in situ chl-*a* values in a log–log transformed scatter plot. Axes were log-transformed since error was proportional to chl-*a* concentration, and the data values spanned several orders of magnitude (Seegers et al., 2018a). For the same reasons, this study used MAE_mult_ as the priority performance metric, which expresses error in terms of the factor by which modeled and observed values tend to differ using a geometric mean (Seegers et al., 2018b):

$${\mathrm{MAE}}_{\mathrm{mult}} = {10}^{\left(\frac{{\sum }_{i=1}^{n}\left|{\mathrm{log}}_{10}\left({M}_{i}\right)- {\mathrm{log}}_{10}\left({O}_{i}\right)\right|}{n}\right)}$$

Terms *M*, *O*, and *n* represent the modeled value, the observation, and the sample size. As an example, a MAE_mult_ value of 1.5 indicates that modeled values are on average 50% different from observed values (in either direction—smaller or larger). Multiplicative bias (bias_mult_) was also used to assess algorithm performance. Bias_mult_ reports values relative to 1, indicating unity, with values < 1 indicating systematic underestimation and values > 1 indicating overestimation.

$${\mathrm{bias}}_{\mathrm{mult}} = {10}^{\left(\frac{{\sum }_{i=1}^{n}{\mathrm{log}}_{10}\left({M}_{i}\right)- {\mathrm{log}}_{10}\left({O}_{i}\right)}{n}\right)}$$

As an example, a bias_mult_ value of 1.2 indicates that modeled values are on average 20% greater than observed values, and a bias_mult_ value of 0.8 indicates modeled values tend to be 20% less than observed values.

### Temporal and spatial analysis

Measures of chl-*a* time series and/or lake-wide gradients are important for water quality managers to assess current water status or condition and may support U.S. Clean Water Act reporting requirements. To illustrate the applicability of the best performing chl-*a* algorithm, time series of in situ and satellite-derived chl*-a* were used to capture within lake variability at multiple discrete sites. Further, spatial composites of chl-*a* enable detection of known chl-*a* gradients and help avoid outlier responses such as erroneously high values along the land–water interface or in different areas of a waterbody (Seegers et al., 2018a, b).

Chl-*a* estimates from the best performing algorithm were compared to field monitoring programs at Lake Champlain—located along the border of Vermont and New York—and at Lake Mendota, Lake Monona, and Trout Lake in Wisconsin. At Lake Champlain, satellite-derived chl-*a* values represent monthly averages (± 1 standard deviation), corresponding to the month of field data collection. At Lake Mendota, Lake Monona, and Trout Lake, satellite-derived chl-*a* values represent daily observations for every date that had a valid satellite observation at each location from April through October. Daily snow and ice data were obtained from the National Snow and Ice Data Center (Urquhart & Schaeffer, 2020). Flags for snow and ice were added to the monthly composites and were developed separately from the Iterative Multisensor Snow and Ice Mapping System Northern Hemisphere Snow and Ice Analysis data (NSIDC, 2008; Version 1.0, 4 km resolution). At each of these lakes, satellite pixels were averaged within a 300-m buffer of the field data collection location.

### Trophic assessment

Trophic assessments may support protection of designated uses, such as fish and aquatic life use, and water quality criteria (Schaeffer et al., 2012, 2013a). Chl-*a* estimates from reliably performing algorithms can complement in situ chl-*a* measures to classify trophic states in inland lakes, flowages, and reservoirs. Therefore, satellite-derived chl-*a* was applied to a subset of resolved lakes that matched Wisconsin Department of Natural Resources (WDNR) sampling in 2018. These lakes were classified by chl-*a* ranges for each trophic category used in the NLA (U.S. EPA, 2009), shown in Table 1. The months of June, July, and August were selected to represent a typical recreational season in the USA that extends from Memorial Day (end of May) through Labor Day (beginning of September). This monthly representation also closely aligned with WDNR seasonal sampling between the target date range of July 15 through September 15, resulting in one sample for each month of July, August, and September (WDNR, 2019). WDNR chl-*a* samples were collected from the top 2 m of the water column at the deepest lake location, or across two to five locations if the lake required additional sampling for characterization. Sample collection, preservation, and storage followed procedures from the WDNR Field Procedures Manual and analysis followed standard methods (Hein, 2017).

**Table 1:** Chl-*a* ranges for each trophic state based on the National Lakes Assessment (NLA)

**Trophic state**	**chl-*a* (µg L**^**−1**^**)**
Oligotrophic	≤ 2
Mesotrophic	> 2 and ≤ 7
Eutrophic	> 7 and ≤ 30
Hypereutrophic	> 30

## Results and discussion

### In situ data availability

**Fig. 3:**
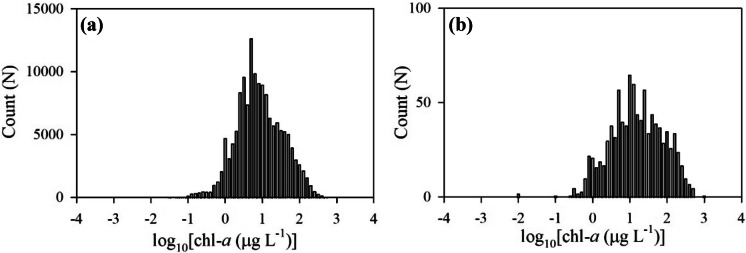
Full distribution **a** of in situ chl-*a* before quality filtering, and the subset distribution of in situ chl-*a* data matched **b** with MERIS and OLCI. Chl-*a* values are reported in log_10_ scale on the *x*-axis

There were 96,707 in situ chl-*a* samples downloaded from the WQP during the MERIS mission from 2002 through 2012, and 61,448 in situ chl-*a* samples during the OLCI mission from 2016 through 2019 (Fig. 3a). Chl-*a* ranged from 0.01 to 2,100,000 μg L^−1^ with a mean of 27.08 μg L^−1^. US lake chl-*a* may range between 0.1 and 1,000 μg L^−1^; Loftin et al. (2016) reported values up to 940 μg L^−1^ across the USA from the 2007 NLA. It may be possible for chl-*a* concentrations to range up to 5,000 μg L^−1^ in cyanobacteria scum conditions or approach 50,000 μg L^−1^ with wind-induced concentrations of scums at the surface (Chorus & Bartram, 1999). Samples above 50,000 μg L^−1^ were rare, only 0.03% of the total samples, and treated with caution as they may be erroneous. However, the filtering criteria removed all these extremely high values from further validation analysis. This removal was likely due to wind advection transporting the scum into land adjacent pixels that were quality flagged to remove straylight contamination. Remaining in situ samples filtered for satellite matches ranged from 0.1 to 872 μg L^−1^ with a mean of 42.2 μg L^−1^ (Fig. 3b). Only 12% of the total samples had a depth measure ≤ 2 m or were labeled “surface” and were retained for satellite match-ups.

**Fig. 4:**
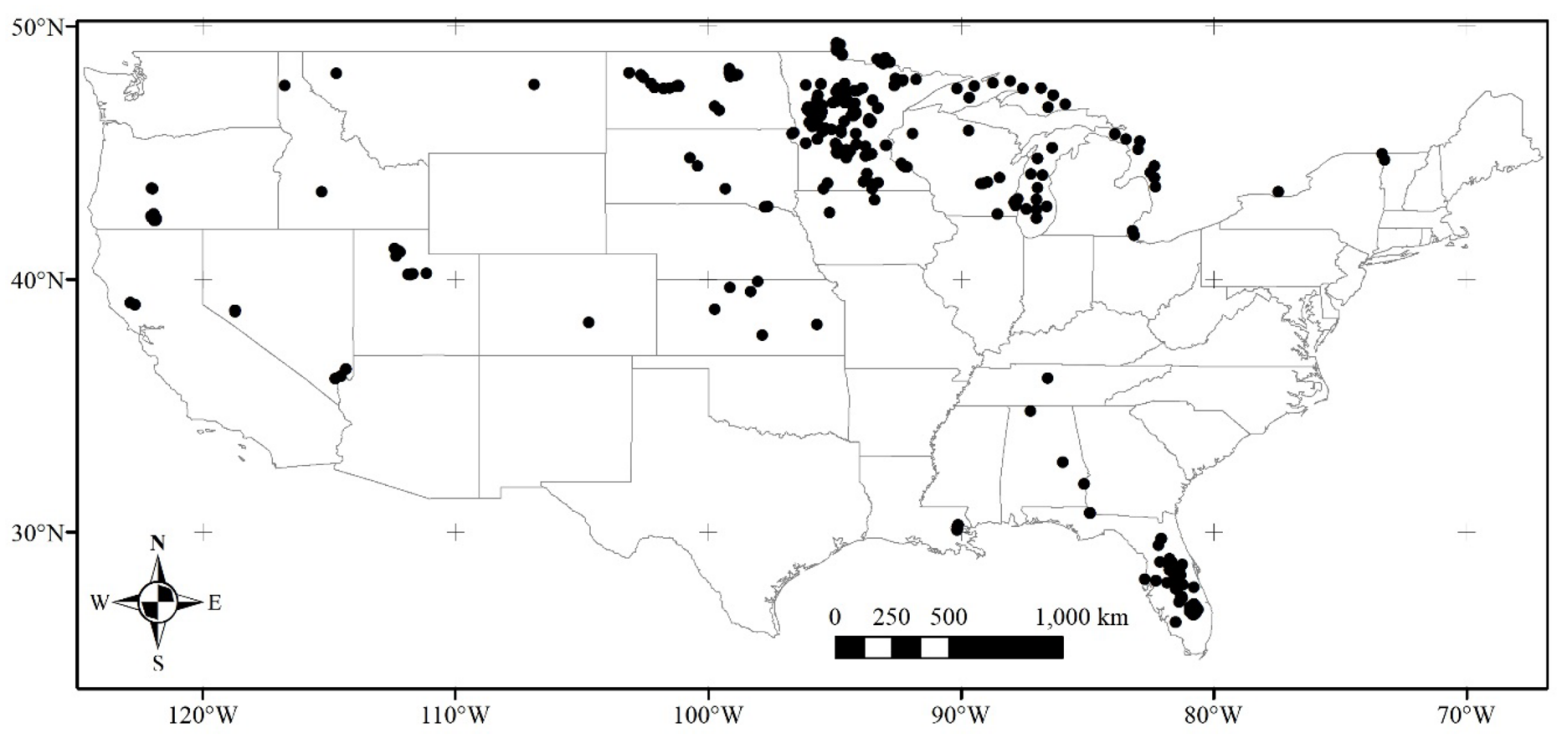
Locations of 181 resolvable inland water bodies for MERIS (2002 through 2012) and OLCI (2016 through 2019) validation from the WQP in situ chl-*a* after quality filtering. MERIS had 946 match-ups, and OLCI had 17 matchups across the continental USA

Spatial filtering to resolvable lakes using the NHD reduced the initial potential matches. Clark et al. (2017) initially identified MERIS and OLCI resolvable lakes from the NHD based on the requirement for a 3 × 3 water pixel array after eliminating adjacent shore pixels. Urquhart and Schaeffer (2020) later updated these findings to lakes that had at least three water pixels remaining in the NHD lake polygons, after quality control flagging pixels adjacent to the shoreline. MERIS and OLCI 300-m at-nadir pixel size limits resolvable lakes in the USA to 0.7% of total lakes as defined by the NHD (Clark et al., 2017). The number of resolvable lakes may fluctuate depending on the resolution of the land mask applied in the satellite processing. Lake shorelines are also fractal (Mandelbrot, 1967), and the resolution of their size is dependent on the method applied to measure the shoreline. In addition, shorelines are dynamic due to flood and drought stages, erosion, and land development (Murray et al., 2019). Validation points are from across 20 of the 50 states in 181 lakes of the 2,370 (7.6%) resolvable lakes (Fig. 4). Of the nine US climate regions (Karl & Koss, 1984), the Upper Midwest had the best validation coverage, while there was minimal representation in the Northeast, Southeast (except Florida), Ohio Valley, South, and Southwest regions. After filtering, there were 946 in situ matches with MERIS and 17 with OLCI representing only 0.6% of the total in situ samples.

### Validation results

**Fig. 5:**
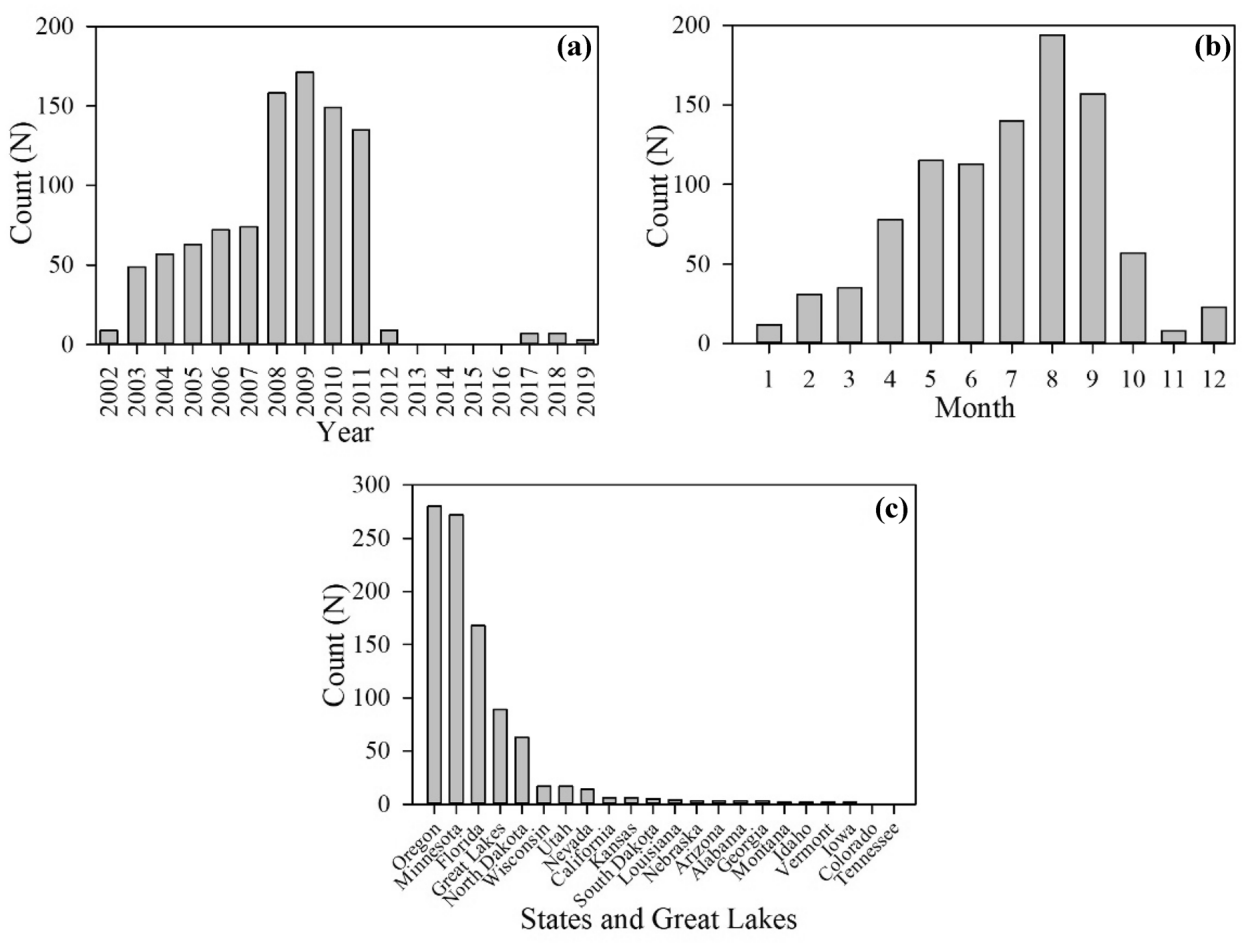
In situ measurement counts matched to the satellite for each **a** calendar year, **b** month, and **c** within states and the Great Lakes

Validation data was over-weighted during MERIS years between 2002 and 2012 compared to OLCI, which had most matches in 2017 and 2018 (Fig. 5a). There was an increase in validation points from 2002 through 2009, a pattern supported by other research reporting a steady increase in WQP chl-*a* data density from 1980 to 2008 (Papenfus et al., 2020). The increase in matchups from 2002 through 2008 was also a result of increased MERIS coverage. MERIS data for North America were obtained by onboard recording prior to 2008. In 2008, the Canadian Centre for Remote Sensing started direct broadcast of MERIS data increasing coverage (Mishra et al., 2019). The lower counts in 2002 and 2012 were a result of partial years from the MERIS sensor, launched in March 2002 and terminating in April 2012. The minimal validation matches for OLCI years may be due to a lag in voluntary reporting to the WQP, a decline in actual in situ monitoring, or some combination thereof. Sampling throughout months of the year was heavily biased toward spring and summer, with the lowest representation in winter months (Fig. 5b). Schaeffer et al. (2018b) and Papenfus et al. (2020) identified similar seasonal in situ sampling biases toward warmer months and under-representation during the coldest months of the year. Most validation locations were from Oregon, Minnesota, Florida, and the Great Lakes (Fig. 5c). Minnesota and Florida are more likely to have matches with OLCI and MERIS because they have some of the highest numbers of chl-*a* records in the WQP and the most resolvable lake observations from satellites (Papenfus et al., 2020).

**Fig. 6:**
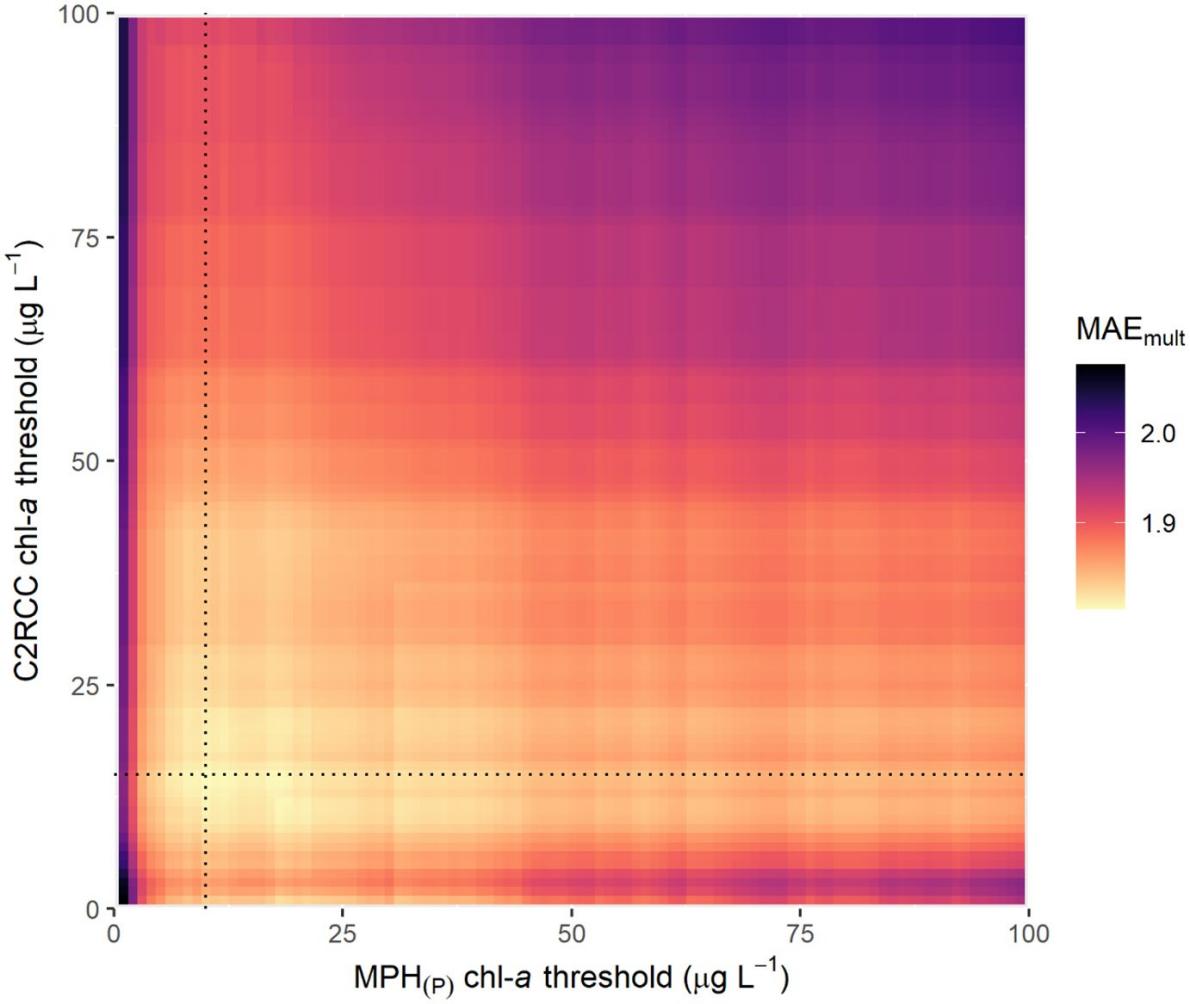
A visualization demonstrating selection of MPH_(P)_ and C2RCC thresholds, showing MAE_mult_ for each possible combination of the two thresholds. If MPH_(P)_ is above the MPH_(P)_ threshold, the algorithm outputs MPH_(P)_; otherwise, it outputs C2RCC, as long as C2RCC is below the C2RCC threshold. The optimal combination of MPH_(P)_ and C2RCC thresholds—i.e., that yielding the lowest MAE_mult_—was selected (10 and 15 µg L^−1^, respectively), and is shown at the intersection of the dotted lines

The analysis to determine the merged algorithm combination with the lowest error yielded a MPH_(P)_ minimum threshold value of 10 µg L^−1^ and a C2RCC maximum threshold value of 15 µg L^−1^, a combination referred to as C_15_-M_10_ (Fig. 6). Thus, the chl-*a* concentration resulting from MPH_(P)_ was retained if chl-*a* was > 10 µg L^−1^; if the chl-*a* concentration was ≤ 10 µg L^−1^, the C2RCC result was selected, as long as the pixel was valid and < 15 µg L^−1^ (see Fig. 2). If the C2RCC result was above 15 µg L^−1^, the output was reported as not a number (NAN), indicating it was invalid. The other two merged algorithms were considered based on natural breaks that occurred in the in situ data set: (1) C_50_-M_10_, with MPH_(P)_ split value at 10 µg L^−1^ and C2RCC chl-*a* maximum threshold at 50 µg L^−1^, and (2) C_50_-M_15_, with MPH_(P)_ split value at 15 µg L^−1^ and C2RCC chl-*a* maximum threshold at 50 µg L^−1^. C_15_-M_10_ was the only merged algorithm exhibiting underestimation bias, though slight. This was because, relative to the other merged algorithms, C_15_-M_10_ was more dominated by MPH_(P)_, which was characterized by a strong negative bias. Conversely, bias_mult_ values for the other merged algorithms were slightly above 1, reflecting the overestimation bias observed for C2RCC.

**Fig. 7:**
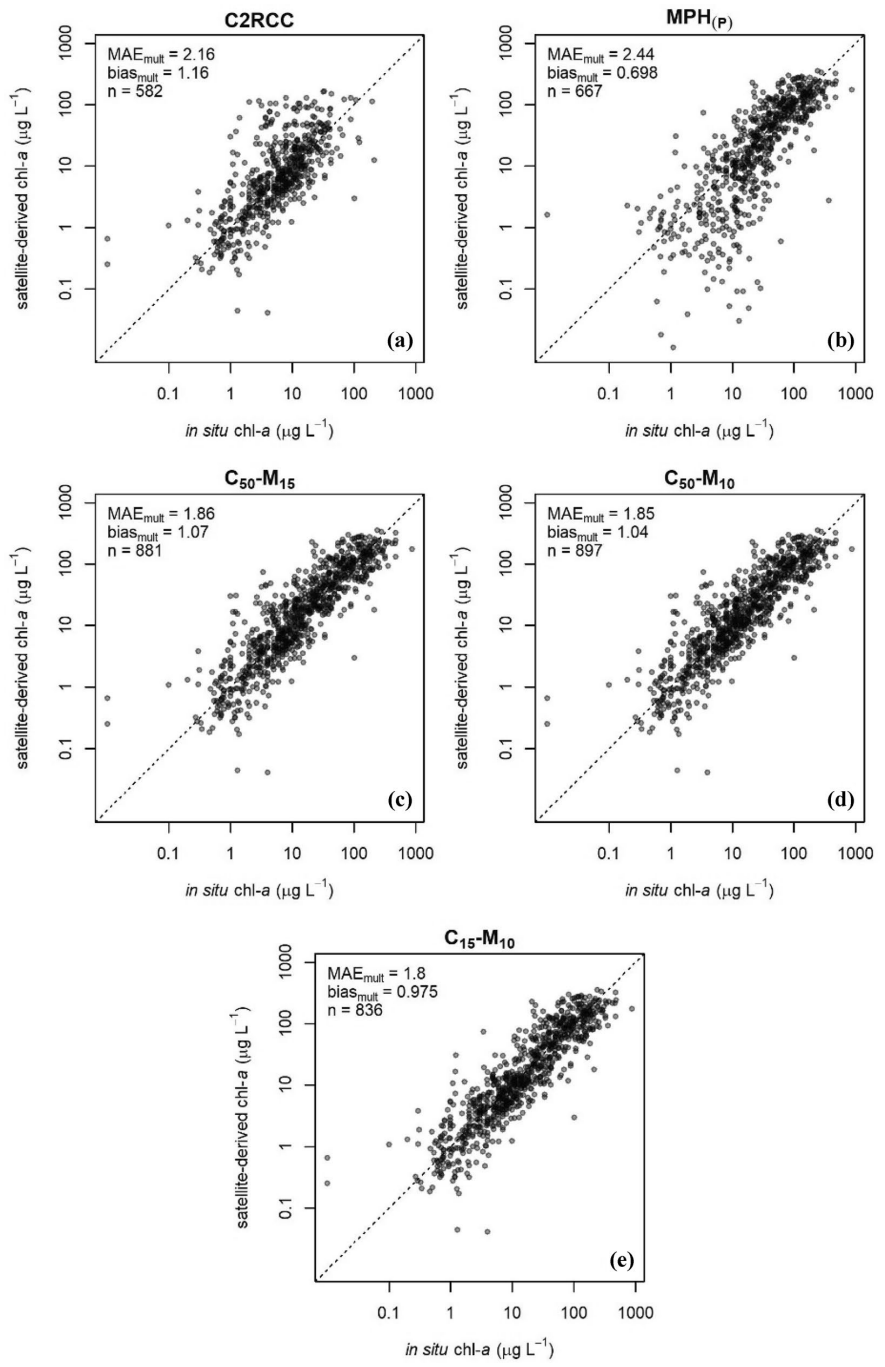
Validation scatterplots for **a** C2RCC, **b** MPH_(P)_, and the three merged algorithms **c** C_50_-M_15_, **d** C_50_-M_10_, and **e** C_15_-M_10_. *n* is the number of validation points. Both axes were log_10_ transformed for display

**Fig. 8:**
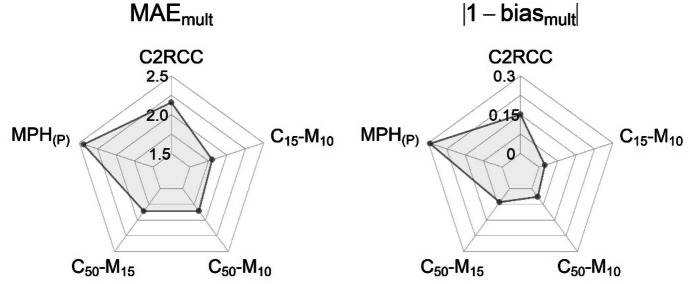
Comparison of the metric results of MAE_mult_ and bias_mult_ summarized in star plots across all five algorithms. The star plot center represents values that indicate best algorithm performance, while farthest from center represents the poorest performance. Here, for visualization purposes, bias_mult_ is displayed as the absolute difference between 1 and bias_mult_ since values further from 1 in either direction indicate a greater bias

The C2RCC, MPH_(P)_, and three merged algorithms were evaluated by comparing their values to coincident in situ chl-*a* samples (supplemental Table S1). Regression plots (Fig. 7) for the five algorithms and performance metrics (Fig. 8) show all three merged algorithms performed better than either the C2RCC or MPH_(P)_ individual algorithms, with notably lower MAE_mult_. Of the five validated algorithms, the merged C_15_-M_10_ algorithm had the lowest MAE_mult_ at 1.80 and smallest bias_mult_ (closest to 1) at 0.975, outperforming all other algorithms in both metrics.

**Fig. 9:**
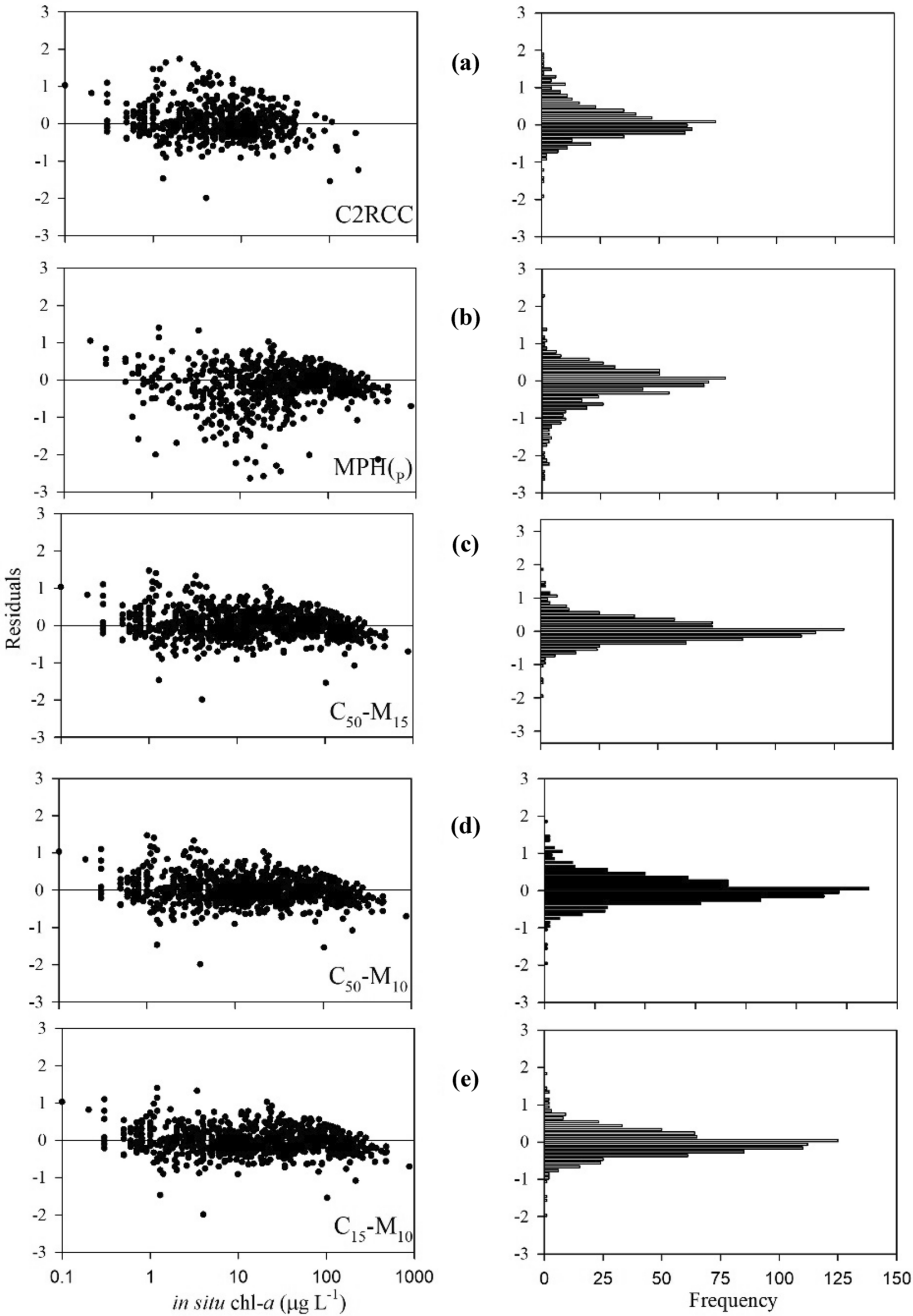
Residual scatterplots and histograms of MERIS and OLCI to in situ chl-*a* matchups. The left panels are residual plots of the difference between model satellite log_10_ chl-*a* and the reference in situ log_10_ values versus reference values. The right panel are histograms of log_10_ summarizing the error distribution of **a** C2RCC, **b** MPH_(P)_, and merged C2RCC-MPH_(P)_ **c** C_50_-M_15_, **d** C_50_-M_10_, and **e** C_15_-M_10_ algorithms

Generally, C2RCC was most effective at low chl-*a* values, as previously reported (Alikas et al., 2010; Giardino et al., 2010), and did not exceed an upper threshold of ~100 µg L^−1^. Log-transformed residuals (Fig. 9) confirmed the relatively even distribution around the unity line, with systematic overestimation at low- to mid-range values, reflected in the bias_mult_ of 1.16, and some degree of underestimation at high values. Conversely, MPH_(P)_ exhibited a strong underestimation bias at low- and mid-range chl-*a* values, while performing reasonably well at high chl-*a* values compared to C2RCC.

The poor performance of C2RCC in highly eutrophic and hypereutrophic US lakes and reservoirs was similar to results across various North American, European, and South African lakes (Binding et al., 2011; Palmer et al., 2015; Kravitz et al., 2020). Where Kravitz et al. (2020) recommended C2RCC only be applied in waters with < 20 μg L^−1^, the 15 μg L^−1^ threshold selected in this study was more conservative. Kravitz et al. (2020) also reported the MPH detection limit was 1–5 μg L^−1^, but the work here showed a heavy bias and increased MAE_mult_ with < 10 μg L^−1^; this difference may be due to the updated MPH index used in this study (Pitarch et al., 2017). This low-end bias and increased MAE_mult_ supported findings from Kravitz et al. (2020) that the MPH_(P)_ algorithm performed most accurately for chl-*a* concentrations > 20 µg L^−1^, when compared to oligotrophic and mesotrophic waters. The differences in the selected algorithm thresholds between this study and previous studies may also be a result of the available distribution of in situ validation points within these ranges.

Validation studies experience limitations and errors in both the in situ measures and satellite data. Single point discrete in situ measures do not represent larger three-dimensional (longitude, latitude, and satellite penetration depth) areas of water, such as a 300 × 300 m pixel from OLCI, especially in heterogenous environments. In situ chl-*a* measures range in error from 30 to 60% (Trees et al., 1985), even though they are frequently considered ground-truth, which is a misnomer. Several other studies have previously reported that fluorometric analyses underestimate chl-*a* values compared to more precise HPLC methods (Kumari, 2005; Pinckney et al., 1994; Welschmeyer, 1994). One confounding factor inherent to satellite remote sensing of inland waters is straylight contamination along the land–water interface (Schaeffer et al., 2012), especially since the shoreline may be a priority management area for recreational purposes; this needs to be addressed in future satellite missions. Here, the nearest two pixels from land were quality flagged, but it has been suggested that up to four pixels from shore may still be under the effects of straylight contamination (Hestir et al., 2015), which would severely limit applications in many smaller water bodies < 0.1 km^2^ (Downing et al., 2006). Satellite-derived bio-geochemical measures also suffer from a lack of standard calibration reference as commonly required with traditional laboratory methods.

### Time series comparison

**Fig. 10:**
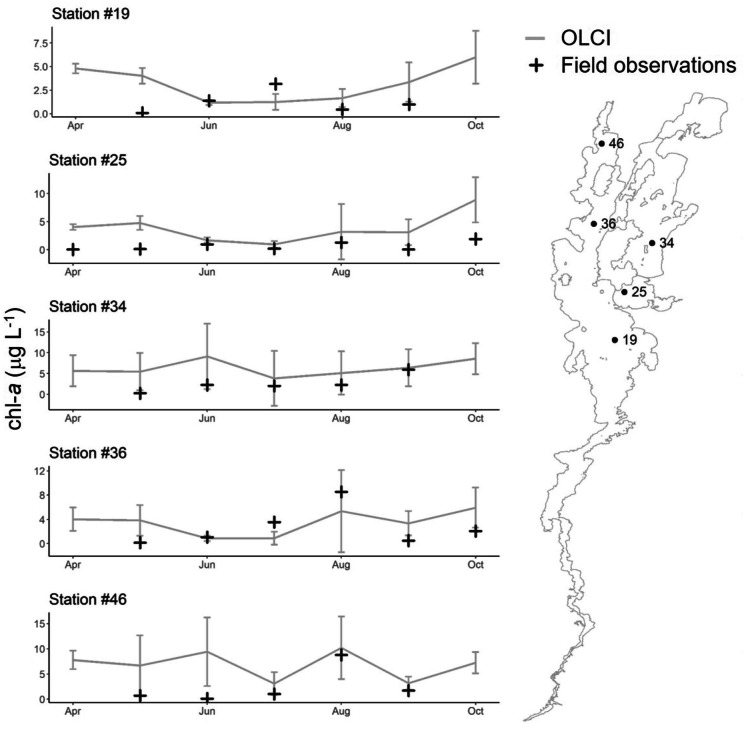
Time series of chl-*a* at five locations across Lake Champlain—located along the border of New York and Vermont—based on satellite observations via the merged C_15_-M_10_ algorithm (gray lines) and using field observations from the Lake Champlain Long-Term Water Quality and Biological Monitoring Project (black points). Satellite observations represent monthly averages (± 1 standard deviation) of all valid satellite overpasses within the month of field data collection and within 300 m of each point sample. Field observations were obtained for a single date each month

At Lake Champlain, the majority of in situ samples were within ± 1 standard deviation of satellite merged C_15_-M_10_ chl-*a* (Fig. 10). Additionally, with the exception of Station #19, field and satellite observations exhibited similar patterns of chl-*a* increase and decrease, despite an offset between the magnitude of field observations and satellite-estimated averages. Generally, in situ observations were lower than satellite-estimated values, which is consistent with the slight bias observed with the merged C_15_-M_10_ algorithm validation (Fig. 8). Some of the bias could be explained by the depth offset between the two datasets; some of the Lake Champlain samples were collected at 1 m below the surface, which may under-represent surface biomass measured by the satellite. Additionally, underestimations have been reported with the in situ fluorometric method (Kumari, 2005; Pinckney et al., 1994; Trees et al., 1985). Mismatches between in situ and satellite measurements could likewise be attributed to temporal offsets in the data: field observations were collected on a single day per month, whereas satellite-derived results shown in Fig. 9 represent average chl-*a* estimates for all valid satellite observations during the corresponding month. Furthermore, a spatial offset exists, as field observations represent a single-point sample, while satellite observations were averaged within a 300-m buffer of each field sample.

Satellite merged C_15_-M_10_ chl-*a* at Lake Champlain were well within the published range. Lu et al. (2016) used field measurements to summarize chl-*a* across the lake for the years 1992 to 2012, finding a range between 0.5 and 40.8 µg L^−1^ with an average of 5.8 µg L^−1^ and a standard deviation of 4.7 µg L^−1^. This study also used satellite imagery from OLCI for the year 2018 at a subset of sites across Lake Champlain, finding a range between 0.8 and 19.3 µg L^−1^ with an average of 4.9 µg L^−1^ and a standard deviation of 3.4 µg L^−1^.

**Fig. 11:**
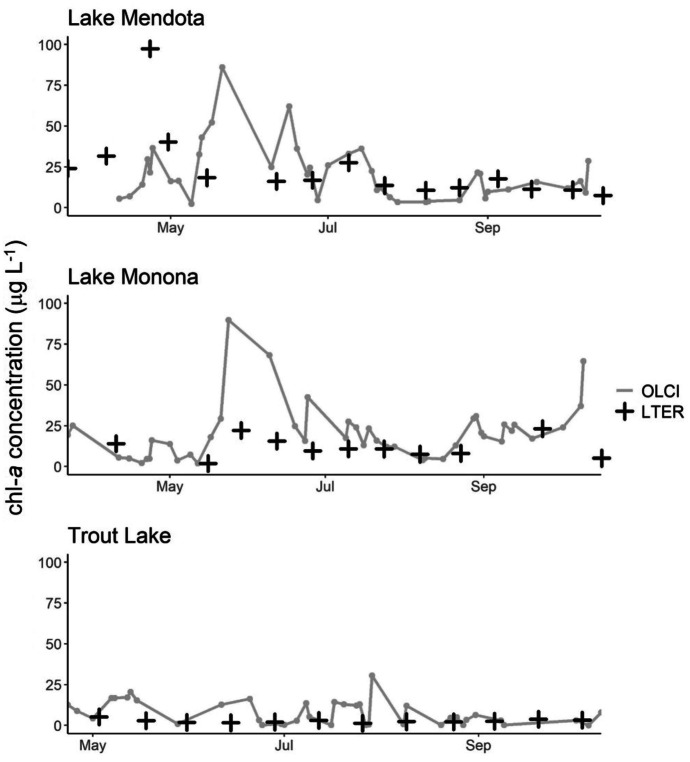
Time series of chl-*a* at three lakes in Wisconsin based on satellite observations via the merged C_15_-M_10_ algorithm (gray lines) and using field observations from the North Temperate Lakes LTER Network. Satellite data represent daily observations averaged within 300 m of each point sample for each date that had a valid satellite observation

LTER in situ measurements at Lake Mendota, Lake Monona, and Trout Lake varied in agreement with satellite estimates (Fig. 11). Visually, measurements at Lake Mendota exhibited the most disagreement, with LTER data indicating a peak in chl-*a* early in the time series and merged C_15_-M_10_ chl-*a* indicating a peak approximately a month later. Later observations demonstrated better agreement, and merged C_15_-M_10_ chl-*a* indicated additional increases in chl-*a* where field observations were not available. These increases, which were not captured through in situ measurements, highlight the increased temporal resolution offered as an advantage of satellite monitoring. At Lake Monona, field measured chl-*a* and satellite merged C_15_-M_10_ chl-*a* followed similar patterns, capturing local minimums and maximums, but differed in their magnitude. Overall, merged C_15_-M_10_ chl-*a* were higher than field measurements, a finding consistent with results presented for Lake Champlain. At Trout Lake, both datasets indicated low chl-*a* concentrations and were closely aligned throughout the entire time series.

At each of the three Wisconsin lakes considered in this comparison, merged C_15_-M_10_ chl-*a* were slightly higher than previously published ranges. Like in Lake Champlain, a satellite bias would be expected since field samples were collected down to 2 m below the surface; this integrated collection that includes lower chl-*a* concentrations at depth, presumably, may under-represent surface concentrations measured by the satellite. The aforementioned fluorescence underestimation may also explain some of the discrepancy. At Lake Mendota, long-term monitoring spanning May to September of 1995 through 2014 suggested an average chl-*a* value of 8.7 µg L^−1^ and at Lake Monona, 10.8 µg L^−1^ (McDonald & Lathrop, 2017). Using merged C_15_-M_10_ chl-*a*, this study found Lake Mendota to range between 2.4 and 86.1 µg L^−1^ with an average of 21.3 µg L^−1^. At Lake Monona, chl-*a* estimates ranged from 2.0 to 89.9 µg L^−1^ with an average of 21.0 µg L^−1^. At Trout Lake, long-term monitoring spanning 1990 through 2014 yielded an average chl-*a* value of 2.6 µg L^−1^ (Jane et al., 2017). Using merged C_15_-M_10_ chl-*a*, this study found Trout Lake to be the lowest of the three sites considered, ranging between 0.05 and 30.6 µg L^−1^ with an average of 7.7 µg L^−1^.

Disagreement between satellite merged C_15_-M_10_ chl-*a* and in situ measurements at Lake Champlain, Lake Mendota, Lake Monona, and Trout Lake can in large part be attributed to mismatches in sampling frequencies between the datasets (Chen et al., 2007b). Additionally, satellite-derived chl-*a* can have several potential sources of error, including contamination from cloud cover, limitations due to snow and ice cover, and potential fluctuations in satellite estimates due to image processing such as atmospheric correction (Harding et al., 2005; Hu et al., 2001). However, satellite imagery likely provides more consistent and frequent observations, both spatially and temporally, acting as an effective complement to field monitoring (Chen et al., 2007b). Chen et al. (2007a) found that multiple observations throughout a single month create more realistic monthly summaries versus using a single value, and seasonal changes can be difficult to discern using a single observation per month. Moreover, satellite imagery provides increased spatial coverage which can reveal spatial patterns not observable through point measurements.

### Application

**Fig. 12:**
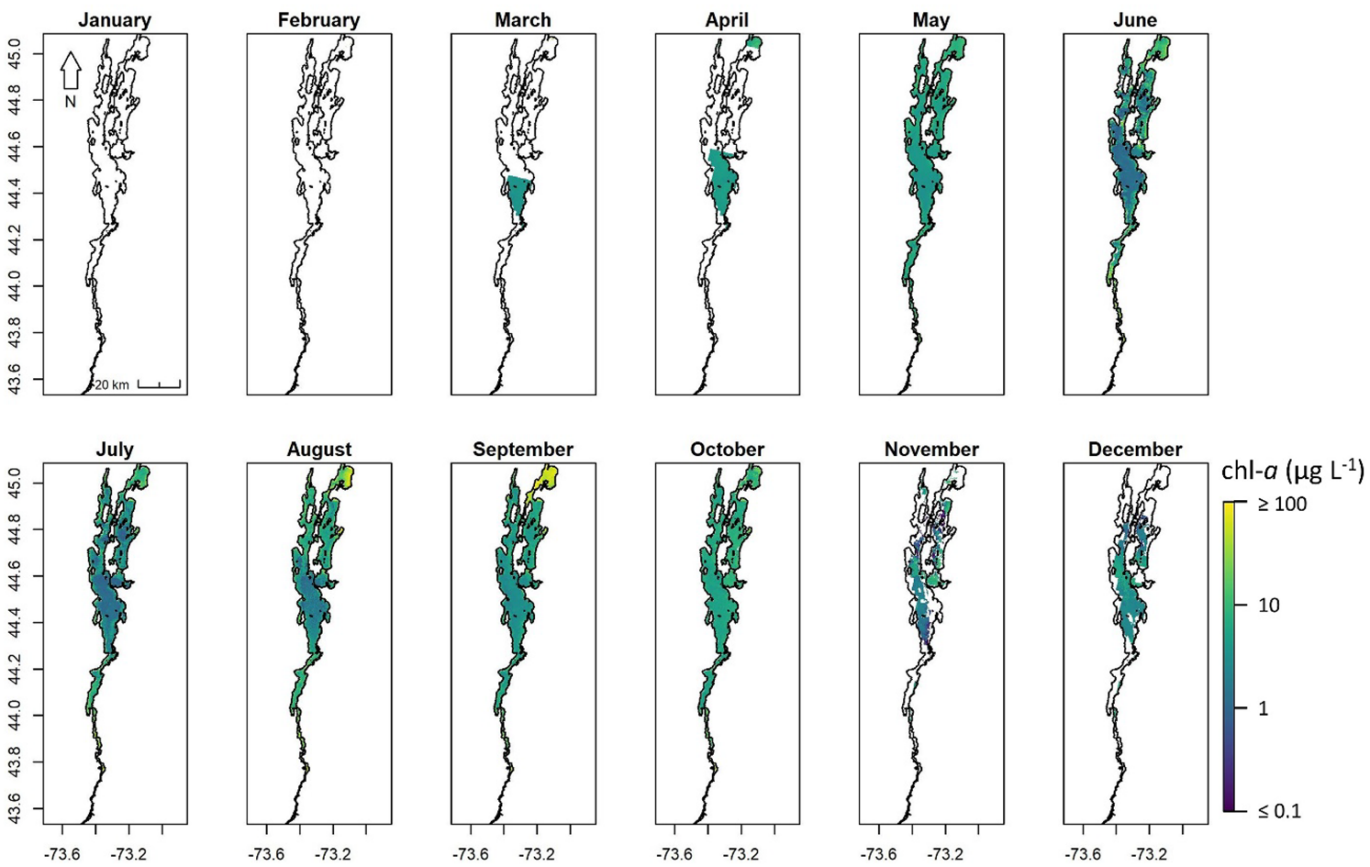
Representative OLCI monthly mean composites of the merged C_15_-M_10_ algorithm chl-*a* in Lake Champlain—located along the border of New York and Vermont—for 2018. White colored pixels inside the lake polygon represent quality flags including mixed pixels, clouds, cloud shadow, snow, and ice

Spatially, the maximum chl-*a* occurred in the northern end of Lake Champlain, Vermont, at Missisquoi Bay with concentrations generally decreasing from north to south (Fig. 12). Temporally, chl-*a* biomass increased from March through April, peaked in September, and declined from October through December. Snow and ice flags and quality control masks limited or completely removed observations from late December through early March. Monthly composites also demonstrate the lack of spatial outliers, particularly along the land–water interface where algorithm saturation due to erroneous straylight could lead to higher errors. The range of chl-*a* from 0 to 25 μg L^−1^ and the north-to-south latitudinal decrease were confirmed by independent measures from the Vermont Department of Environmental Conservation long term monitoring project (VT DEC, 2020), in which the 1992–2019 chl-*a* distributions ranged from 0 to 35 μg L^−1^. The median long-term range in Missisquoi Bay was between 10 and 15 μg L^−1^, with the upper quartile between 20 and 25 μg L^−1^ and the 90th percentile at 35 μg L^−1^. This demonstrates a potential application of the C_15_-M_10_ algorithm within a single waterbody over a diverse range of concentrations within an optically complex system.

**Fig. 13:**
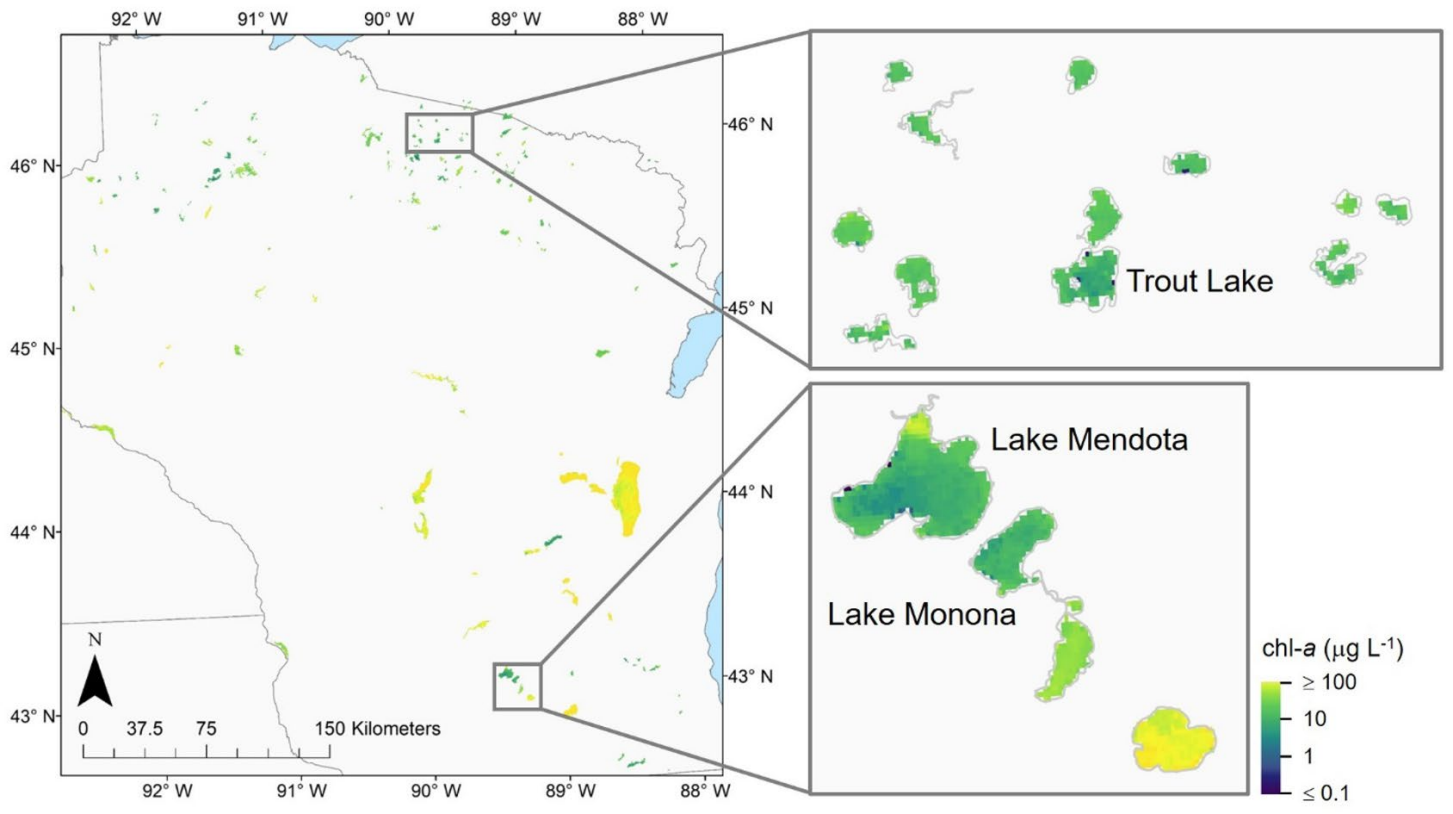
Representative average log_10_ chl-*a* for the state of Wisconsin in August 2018 based on results from the merged C_15_-M_10_ algorithm. A total of 138 lakes in Wisconsin were viewable to provide a means of assessing algorithm behavior and consistency in time and space. The satellite imagery can be used to evaluate algorithm spatial extent of valid retrievals, temporal consistency in retrievals, and spatiotemporal distributions of error metrics from compiled satellite pixels. Subsets show pixel-level results at several lakes including Lake Mendota, Lake Monona, and Trout Lake, where LTER data was compared to satellite estimates

There are an estimated 15,000 lakes within the State of Wisconsin, where Sentinel-3 OLCI resolved 138, or 0.92%, of the largest lakes (Schaeffer et al., 2018a). Past remote sensing efforts in the state included the use of the Landsat missions to complement trophic state assessments (Greb et al., 2009) and studies of water quality patterns across the state. Northern lakes were found to be highly affected by colored dissolved organic matter with greater water clarity, while southern lakes showed an increased influence by algae and suspended sediments due to more intense agricultural land use (Rose et al., 2017). Spatially (Fig. 13), the C_15_-M_10_ algorithm followed a comparable pattern, with northern lakes generally low in chl-*a* and central and southern lakes higher in chl-*a*. LTER subsets indicate little variability in chl*-a* except for slightly increased chl-*a* in the Yahara River estuary, the primary inlet of Lake Mendota located in the northeastern part of the lake, where most suspended sediments reach the lake (Wu et al., 2013).

**Fig. 14:**
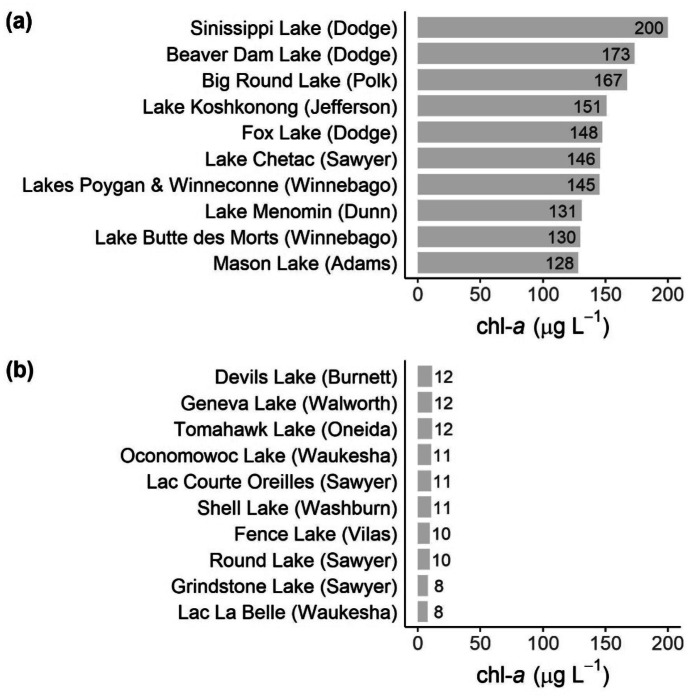
Average chl-*a* at the ten satellite resolved lakes with the **a** highest and **b** lowest chl-*a* for the state of Wisconsin in August 2018 based on results from the merged C_15_-M_10_ algorithm. The county names are provided in parentheses. This serves as an application example for the potential use of merged C_15_-M_10_ algorithm chl-*a* to support assessments of lake health

The merged C_15_-M_10_ algorithm appositely identified some of the lakes and reservoirs with exceptionally high (Fig. 14a) and low (Fig. 14b) chl-*a* through a statewide ranking process. Beaver Dam Lake (Dodge County, Wisconsin), a shallow lowland reservoir with a size of 2591 ha and a mean depth of only 1.5 m, has experienced significant summer algal blooms in the past, and its health is listed as poor (WDNR, 2020). It was assessed in 2012 and remains listed as impaired as total phosphorus and chl-*a* exceed the Wisconsin Consolidated Assessment and Listing Methodology (WisCALM) thresholds for Fish and Aquatic Life as well as Recreation Use. The satellite-derived chl-*a* value of 173 µg L^−1^, which was the 2nd highest for the Sentinel-3 OLCI resolved lakes in the state in August 2018 (Fig. 14a), was within the chl-*a* range of 27.7–393 µg L^−1^ measured for this lake from 2016 to 2020 and indicated a moderate summer algal bloom.

This is in stark contrast to Shell Lake (Washburn County, Wisconsin), a shallow seepage lake with a size of 1017 ha and a mean depth of 7.0 m. The health of this lake is listed as excellent. Shell Lake was assessed in the 2016 listing cycle and is currently not considered impaired as total phosphorus and chl-*a* do not exceed the WisCALM thresholds for Fish and Aquatic Life and Recreation Use. The satellite-derived chl-*a* value of 11 µg L^−1^, which was the 4th lowest in August 2018 (Fig. 14b), matched the chl-*a* range of 0.5–22.9 µg L^−1^ measured from 2016 to 2020. However, the merged C_15_-M_10_ chl-*a* were two to four times higher than previously measured ranges for seven of the lakes. This does not necessarily mean the satellite over-estimated but measured more of the lake spatially and temporally than would be achieved through field monitoring, so it was possible past field observations were unable to capture the highest concentrations.

**Fig. 15:**
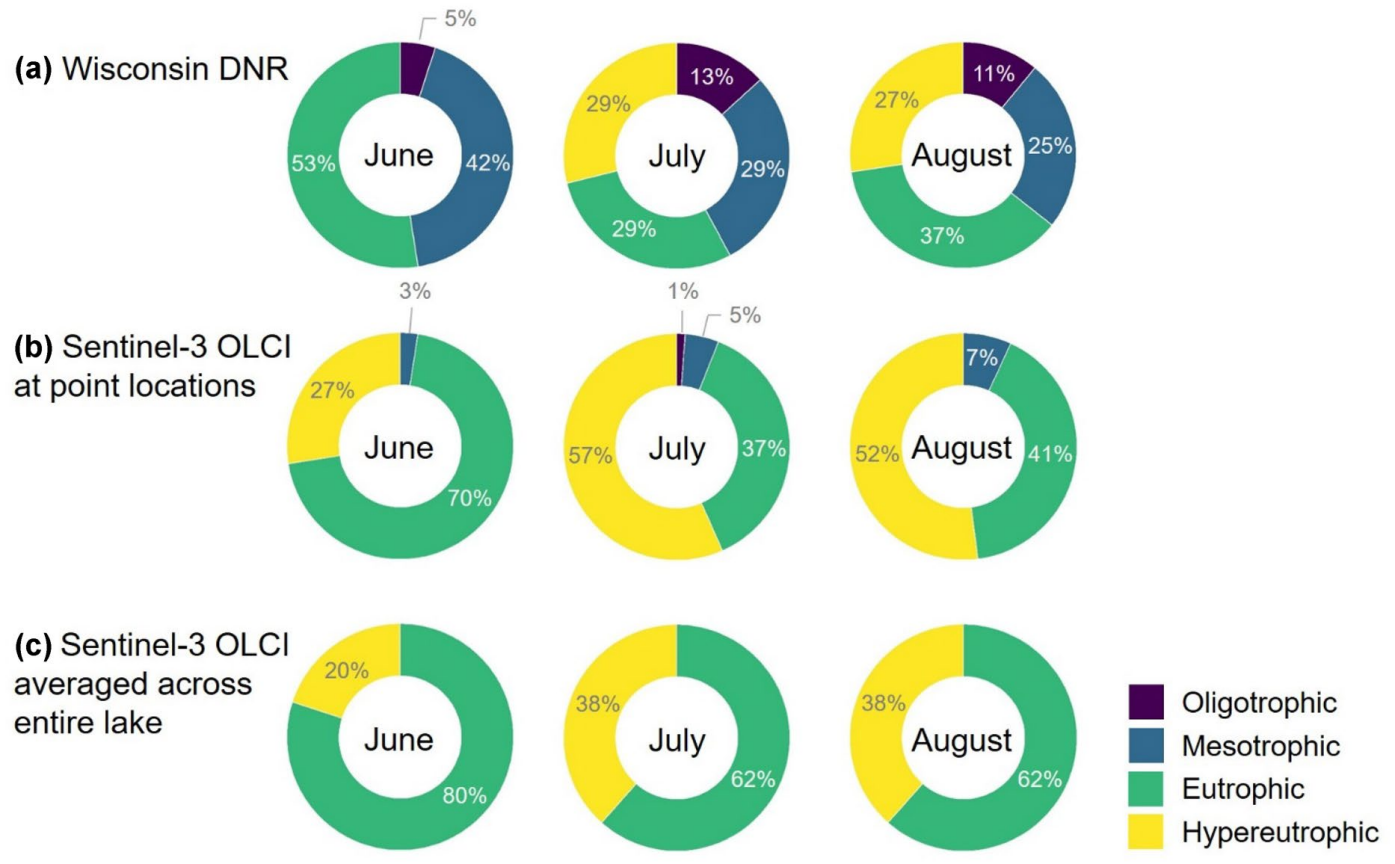
Trophic states for a subset of satellite resolved lakes where WDNR chl-*a* samples were collected in June, July, and August 2018. Trophic states were deduced from **a** WDNR chl-*a* samples, **b** the merged C_15_-M_10_ algorithm at the respective point locations, and **c** the merged C_15_-M_10_ algorithm averaged across the entire lake. This serves as an application example for the statewide quantification of trophic states in Wisconsin and can be rescaled for any number of lakes or reservoirs

These relatively high C_15_-M_10_ chl-*a* values are reflected in the trophic states of a subset of satellite resolved lakes. WDNR chl-*a* samples collected from June to August 2018 indicate a decrease in the number of combined oligotrophic and mesotrophic lakes from 47% in June to 36% in August 2018 (Fig. 15a; supplemental Table S2). The C_15_-M_10_ algorithm indicates an increase from 3% in June to 7% in August 2018 at the respective point locations (Fig. 15b; supplemental Table S2). Again, the bias in the algorithm may result from the WDNR sample collections down to 2 m depth either potentially not capturing the surface biomass or including lower concentrations at depth, and mismatches in the exact timing and dates of the in situ sampling versus satellite measures. The merged C_15_-M_10_ algorithm averaged across the lake indicates 0% oligotrophic and mesotrophic lakes in the summer months (Fig. 15c), which can be attributed to the lack of spatial representativeness of the point locations. This difference between the satellite and in situ point locations with averages across the entire lake is another example where the spatial and temporal coverage of satellites may complement single monthly measures at fixed monitoring locations. The difference between the satellite point location results (Fig. 15b) and averages across lakes (Fig. 15c) addresses a challenge in the use of water quality indicators by providing flexibility in the definition of spatial and temporal scales for which the indicator is relevant (Bierman et al., 2011; Rees et al., 2008). Satellite water quality monitoring methods provide the option to report chl-*a* both at a fine spatial resolution, such as at point locations, and across a broad spatial scale, such as averaged across lakes, which is relevant for management applications and decision-making efforts. In situ measures do not provide the same flexibilities temporally and spatially. In the case of lakes, nutrients are not limited to impacting the single point locations within a system, but impact the entire system (Guildford & Hecky, 2000). These results demonstrate the algorithm application, while biased, could still support assessments of lake health through the identification of lakes with exceptionally high and low chl-*a* and address some of the challenges in the use of remote sensing data for the statewide quantification of trophic states in Wisconsin (Greb et al., 2009).

## Conclusion

Chl-*a* serves as a proxy for phytoplankton biomass and is an ecologically important indicator of aquatic ecosystem health and condition. The C2RCC chl-*a* retrieval algorithm, MPH_(P)_ algorithm, and three merged scenarios were assessed. The best performance based on mean absolute multiplicative error (MAE_mult_) was from the merged algorithm referred to as C_15_-M_10_. Validation occurred across 20 of the 50 states in 181 lakes of the 2,370 resolvable lakes. This study contributes to the transition of chl-*a* algorithm maturity (NASA, 2020) from stage 1 which consists of quantifying error statistics on a small number of measurements from selected locations and times toward stage 2 which consists of assessing the algorithm across a number of locations and times; with some initial convergence of findings with similar efforts. However, more effort would be required to complete the transition and continue to advance maturity levels, as this is the first study to examine the merged results of two algorithms previously only independently assessed. Satellite-derived measures were demonstrated to complement in situ water quality time series in Lake Champlain and spatially across Wisconsin lakes within previously published ranges even with a slight bias. The combination of satellite measures and in situ data will allow for more frequent reporting than otherwise possible with field sampling alone. Further, the value of satellite-derived chl-*a* was demonstrated to adequately classify Wisconsin resolvable lakes for trophic state assessments. Continued demonstration and convergence of algorithm performance evidence may allow for these satellite measures to eventually be considered by more management agencies in assessments and reporting, such as WDNR’s use of the Landsat missions to complement trophic reporting (WDNR, 2019).

## Supplementary Information

Below is the link to the electronic supplementary material.

Supplementary file1 (XLSX 98 KB)
